# Research Progress on the Photo-Driven Catalytic Production of Biodiesel

**DOI:** 10.3389/fchem.2022.904251

**Published:** 2022-04-25

**Authors:** Jinshu Huang, Yumei Jian, Ping Zhu, Omar Abdelaziz, Hu Li

**Affiliations:** ^1^ State Key Laboratory Breeding Base of Green Pesticide and Agricultural Bioengineering, Key Laboratory of Green Pesticide and Agricultural Bioengineering, State-Local Joint Laboratory for Comprehensive Utilization of Biomass, Ministry of Education, Center for R&D of Fine Chemicals, Guizhou University, Guiyang, China; ^2^ Department of Chemistry, Centre for Catalysis and Sustainable Chemistry, Technical University of Denmark, Kemitorvet, Denmark; ^3^ Department of Chemical Engineering, Lund University, Lund, Sweden

**Keywords:** biomass, biodiesel, biofuels, photocatalysis, photo-driven catalysis

## Abstract

Biodiesel considered a green, environmentally friendly, and renewable energy source is one of the most promising candidates to replace fossil fuels to supply energy for the world. The conventional thermocatalytic methods have been extensively explored for producing biodiesel, while inevitably encountering some drawbacks, such as harsh operating conditions and high energy consumption. The catalytic production of biodiesel under mild conditions is a research hotspot but with difficulty. Photocatalysis has recently been highlighted as an eco-friendly and energy-saving approach for biodiesel production. This mini-review summarizes typical photocatalysts for biodiesel production and discusses in detail the catalytic mechanism and strategies of the photo-driven (trans)esterification to produce biodiesel. The current challenges and future opportunities of photo-driven catalysis to prepare biodiesel are also outlined, in steps towards guiding the design of advanced photocatalysts for biodiesel production.

## Introduction

With the rapid development of global industrialization, the total global fossil energy consumption is expected to increase by 28% between 2015 and 2040, resulting in a gradual reduction of fossil energy stocks (Manique et al., 2016; Wang A et al., 2018; Li et al., 2019a; Ghani et al., 2021). In the meantime, people’s awareness of environmental protection and the growing energy demand are stimulating the development enthusiasm of countries around the world to seek renewable energy (Zhang et al., 2019a; Zhao et al., 2019; Guo et al., 2021; Umar et al., 2022). Biodiesel, as a clean and renewable energy with low sulfur content, stands out in many renewable energy sources and is gradually widely used. It has long been regarded not only as a sustainable green fuel, but also as a raw material or intermediate for the synthesis of fine chemicals (e.g., industrial solvents, surfactants, and lubricants) (Zhang et al., 2019a; Zhang et al., 2019b). The carbon dioxide produced in the combustion process can be recycled through photosynthesis to slow down the greenhouse effect, which has good environmental protection performance (Karimi Estahbanati et al., 2020). Biodiesel has the advantages of good combustion performance, good low temperature starting performance, excellent lubrication performance, and high safety performance, which can effectively reduce the wear rate of engine parts and prolong the service life of the engine (Zhao et al., 2019; Guo et al., 2021). Biodiesel production has been continuously increasing over the past years and is anticipated to increase further in the future (Ahmed et al., 2016). In 2008, approximately 36.8 million liters of biodiesel were produced worldwide, and its total production is estimated to reach approximately 44 million liters in 2027 (Zhang et al., 2019b; Karimi Estahbanati et al., 2020). Overall, biodiesel is one of the most promising candidates for replacing fossil fuels to power the world.

As a rich, clean, and renewable energy source, solar energy is widely used in various fields, including wastewater treatment, solar evaporation, photoelectric treatment, and photothermal catalysis (Li et al., 2020; Nishiyama et al., 2021; Meng et al., 2022; Song et al., 2022). In recent years, the utilization of solar energy to enable heterogeneous catalytic reactions of fuels and chemicals has received extensive attention as a promising alternative to conventional thermal-driven heterogeneous catalytic processes (Liu et al., 2015; Meng and Li, 2021; Guo et al., 2022; Song et al., 2022). Among them, solar-driven (trans)esterification of vegetable oils (e.g., *Jatropha*, saffron, semen, and rapeseed oils), animal oils (e.g., tallow and lard), and edible waste oils or microalgae oils with a short-chain alcohol (e.g., methanol an ethanol) to prepare biodiesel has attracted widespread attention (Corro et al., 2017; de Medeiros et al., 2020; Ghani et al., 2021; Guo et al., 2021), considering its high efficiency, environmental protection, energy-saving feature, and simple operation. However, most photocatalytic materials can achieve higher catalytic efficiency only under ultraviolet light irradiation, and the absorption of sunlight in the visible and infrared regions is very weak, which limits the full utilization of solar energy (Li et al., 2019b; Guo et al., 2022; Song et al., 2022). In addition, photocatalyst has the problem of low separation efficiency of photogenerated carriers (electrons and holes) (Meng et al., 2022). Therefore, it is urgent to develop photocatalysts with a strong visible light response and high charge carrier separation efficiency (Corro et al., 2017; Aghilinategh et al., 2019; Song et al., 2022). At present, morphology control, crystal surface control, energy band control, metal and non-metal doping, preparation of carbon-containing composites, and the formation of heterojunction are mainly used to enhance the photocatalytic performance of photocatalysts (Li et al., 2015a; Wang L et al., 2018).

In the past few years, many excellent reviews have discussed biodiesel production from different aspects of using various thermal catalysts (Ma and Hanna, 1999; Hoekman et al., 2012; Wang et al., 2020; Zhang et al., 2020; Tan et al., 2021; Wang et al., 2021). However, there is a lack of discussion on the photocatalytic production of biodiesel. In this mini-review, the research progress on the production of biodiesel from plant/animal oils and free fatty acids (FFAs) over photocatalysts under light irradiation is summarized, and the mechanism of photocatalytic (trans)esterification and three typical enhanced photocatalytic activity strategies are discussed in detail, e.g., enhancing light absorption, improving the separation and transmission of photogenerated carriers, and promoting the interface reaction.

## Mechanism of Photocatalytic (Trans)esterification

**FIGURE 1:**
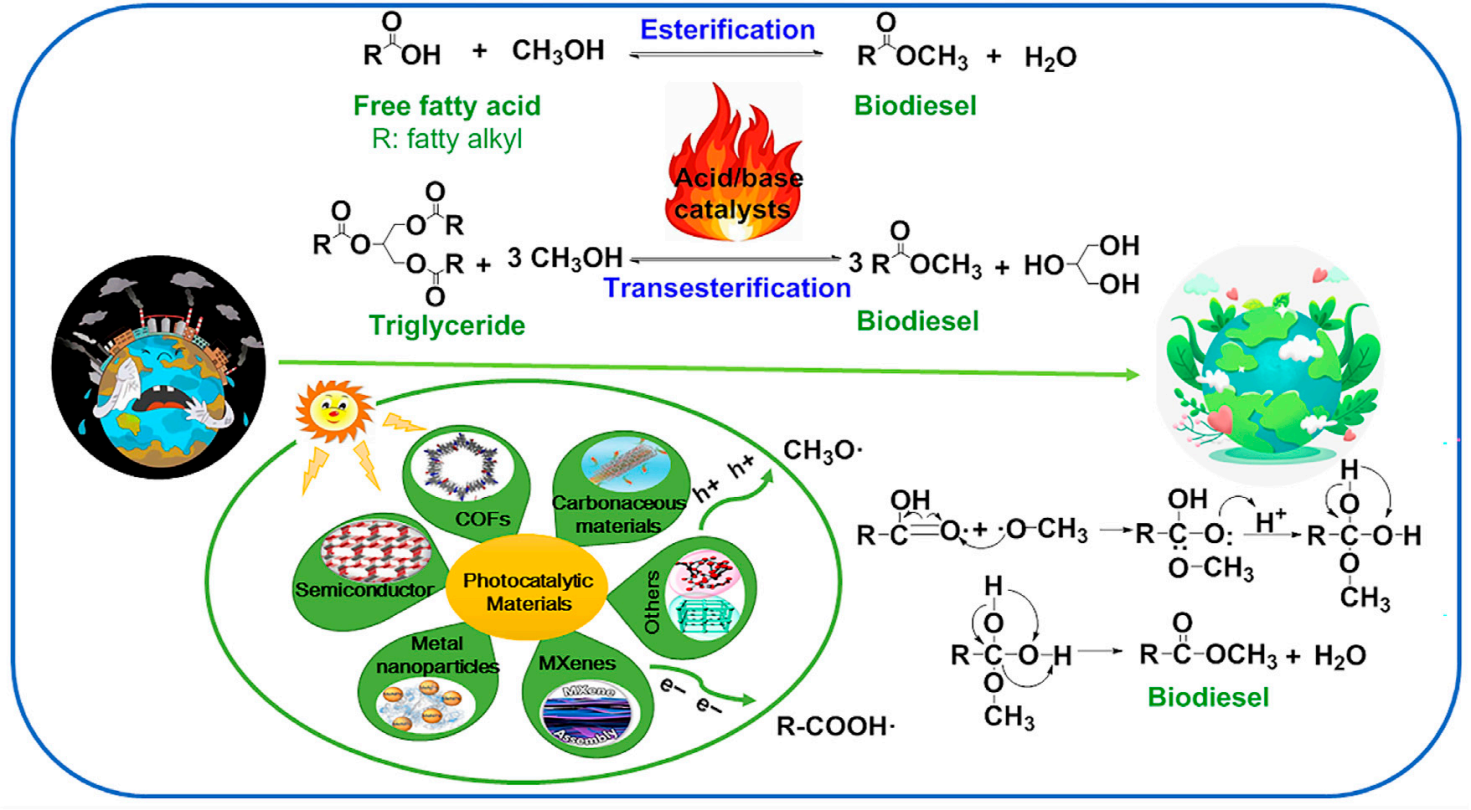
Schematic illustration of thermal- and photo-driven biodiesel production from FFA using acid/base catalysts and photocatalytic materials. Adapted with permissions from Corro et al., 2017; de Medeiros et al., 2020; Ghani et al., 2021; Guo et al., 2021.

Under light irradiation, the photogenerated electrons (e^−^) migrate from the valence band (VB) to the conduction band; at the same time generating the same number of photogenerated holes (h^+^) on the VB. Photocatalytic (trans)esterification to produce biodiesel follows the Langmuir–Hinshelwood reaction path and can be divided into four steps (Corro et al., 2017; de Medeiros et al., 2020; Ghani et al., 2021; Guo et al., 2021) (Figure 1). The production of biodiesel from FFAs and methanol was taken as an example. In the first step, methanol (CH_3_OH) was adsorbed on the surface of the photocatalyst and combined with photogenerated holes to form methanol free radicals (CH_3_O⋅), and then FFAs (R-COOH) and photogenerated electrons generate R-COO⋅. In the second step, the methanol radical (CH_3_O⋅) attacks the carbonyl carbon on R-COOH⋅ to form intermediates. In the third step, fatty acid methyl ester, namely biodiesel, was obtained by intermediate rearrangement dehydration. In the fourth step, the product was desorbed from the photocatalyst and separated into the liquid phase. It is worth noting that both methanol radical (CH_3_O·) and R-COOH· are generated, which is attributed to the simultaneous generation of photogenerated electrons and holes under light irradiation. In addition, vigorous stirring in the process of reactant adsorption and product desorption is conducive to the reaction, which is attributed to the fact that stirring can accelerate the transfer rate of reactants and products at the interface and liquid phase.

### Metal Nanoparticles

Photocatalytic materials have developed rapidly in recent years. Different photocatalytic materials have different catalytic effects on different reactions. It is of great significance to design reasonable photocatalytic materials according to the catalytic reaction. Therefore, this section introduces the different types of photocatalytic materials and their research in the catalytic production of biodiesel.

Metal nanoparticles can effectively absorb solar energy and drive various chemical reactions. Its significant advantage is that their light absorption is not limited to specific wavelengths, which can effectively improve the utilization of solar energy (Li et al., 2017a; Han et al., 2018; Song et al., 2022). Metal nanoparticles generate surface plasmon resonance (SPR) effect after absorbing radiation of specific wavelengths, which causes strong absorption of light and it determines the performance of metal nanoparticle photocatalysts. It was found that copper (Cu), iron (Fe), nickel (Ni), gold (Au), silver (Ag), and other metal nanoparticles can produce the SPR effect and have strong absorption of visible light. The morphology, size, composition, and interaction between nanoparticles will affect the absorption of light, thereby affecting its photocatalytic performance (Li et al., 2017b; Han et al., 2018; Song et al., 2022). This means that the above factors can be adjusted to design and prepare metal nanoparticles photocatalyst with high solar energy utilization. Compared with conventional thermal-driven (trans)esterification for biodiesel production, photo-driven metal nanoparticle catalysts are more energy-saving, environmentally friendly, and efficient due to the absence of heating in both reactors and solvents. Although metal nanomaterials have excellent photocatalytic properties, they are prone to agglomeration, poisoning, and high cost, resulting in low carrier separation efficiency and atom economy.

To solve the above problems, Corro et al. reported that Cr was loaded on SiO_2_ to prevent the aggregation of Cr, which can improve its photocatalytic performance. The catalytic activity of 1% Cr/SiO_2_ catalyst for the production of biodiesel from frying waste oil was excellent and remained unchanged after being repeated use 10 times (yield: 20%) (Corro et al., 2017). Nagaraju et al. also adopted the same method to prepare Ag/ZnO photocatalysis by loading Ag on ZnO for the transesterification of Simarouba oil to produce biodiesel with the yield of up to 84.5% (Nagaraju et al., 2017). Although these photocatalysts have good catalytic performance on oils or ester, they are noble metals or heavy metals. Therefore, Haq et al. prepared SiO_2_-Cu@Fe_2_O_3_ photocatalyst with hexadecyl trimethyl ammonium bromide as a surfactant, which could efficiently catalyze the conversion of edible waste oil into biodiesel (yield: 98%) (Ul Haq et al., 2021). Compared with the reported catalysts, SiO_2_-Cu@Fe_2_O_3_ had superior photocatalytic performance, low preparation cost, and safety, but the preparation method is complex. As such, the photocatalysis of metal nanoparticles for the industrial production of biodiesel still faces challenges.

### Semiconductors

Semiconductor photocatalysts, containing metal oxides (e.g., ZnO and TiO_2_), nitrides or sulfides (e.g., CdS and MoS_2_), and metal-free semiconductors, are widely used in various photocatalytic reactions due to their advantages of simple preparation, low cost, low toxicity, and adjustable band gap (Li et al., 2013; Weng et al., 2019; Dou et al., 2021; Hassani et al., 2021). The application of semiconductor photocatalyst in the catalytic production of biodiesel has been widely studied and high yield has been obtained (Aghilinategh et al., 2019; Ghani et al., 2021; Guo et al., 2021; Nadeem et al., 2021; Guo et al., 2022). Manique et al. used TiO_2_ as a photocatalyst to catalyze the esterification of oleic acid to prepare biodiesel with a high yield of 86% (Manique et al., 2016). However, TiO_2_ has a wide band gap (3.28 eV), and only UV light can be absorbed to effectively generate photogenerated carriers. There is no obvious absorption of infrared and visible light in sunlight, of which ultraviolet (UV) light (>780 nm) accounts for 4% of sunlight and infrared (>780 nm) and visible light (400–780 nm) account for 96% of sunlight, resulting in the low utilization rate of sunlight. Singh et al. reported that the introduction of ZnO with a similar band structure (E_g_ = 3.2 eV) into TiO_2_ inhibited the growth of TiO_2_ particles, improved the optical absorption, and promoted the separation of photogenerated carriers by forming doping levels, which could increase the photocatalytic activity of TiO_2_ (Singh et al., 2020). Corro et al. prepared ZnO/SiO_2_ photocatalyst to catalyze the production of biodiesel from *Jatropha curcas* oil, and the yield reached 96%. After 10 times of recycling, the catalytic activity remained basically unchanged (Corro et al., 2013). In addition, Cao et al. prepared N-doped TiO_2_ photocatalyst by doping non-metallic method had narrow band gap and significantly enhanced absorption of sunlight compared with ordinary TiO_2_, (Cao et al., 2021). Guo et al. reported that doping rare-earth ions La^3+^ in TiO_2_ could promote the separation of photogenerated carriers due to the formation of oxygen vacancies (Guo et al., 2021). In addition to the above methods for modifying the photocatalytic performance, the photocatalytic performance of TiO_2_ can be improved by changing its morphology and forming a local electric field. In short, there is a lack of reports on semiconductor photocatalyst for biodiesel production.

### Covalent Organic Frameworks (COFs)

Covalent organic frameworks (COFs) are polymers with clear two-dimensional and three-dimensional structures. It is applied in various fields (e.g., gas storage, heterogeneous catalysis, energy storage, organic electronic devices, and degradation of pollutants), which is attributed to its advantages of low density, high specific surface area and pore size, and stable porosity (Li H et al., 2021; You et al., 2021; Guo et al., 2022). COF-based photocatalysts with unique photocatalytic properties can be designed in advance by adjusting their topological structure. In recent years, COF materials have been developed rapidly as photocatalysts for various chemical reactions, including photocatalytic water decomposition to produce hydrogen and oxygen, photocatalytic degradation of pollutants, and carbon dioxide reduction engineering (Li H et al., 2021; Li X et al., 2021; You et al., 2021). At present, the amount of COF-based photocatalyst synthesized in the laboratory is relatively small, which is generally prepared at the milligram level, attributed to the harsh synthesis conditions (e.g., high preparation temperature, high energy consumption, and time-consuming) and poor crystallinity of the product. So, it seems not suitable for large-scale production. Based on the above problems, there are limited studies on COF-based catalysts for the catalytic production of biodiesel. In 2021, Zhou et al. synthesized the core-shell magnetic Fe_3_O_4_@COF-OMe catalyst at room temperature (Zhou et al., 2021). Due to its high specific surface area, porosity, and good chemical stability, the thermal-driven catalytic synthesis of biodiesel from *Jatropha* oil was successfully realized, and the yield was about 70%. However, there is no report on COF-based photocatalytic production of biodiesel. Therefore, it is of great significance to develop COF-based photocatalysts with high photocatalytic activity for the catalytic production of biodiesel.

### Carbonaceous Materials

Carbonaceous materials are promising catalytic materials with various forms, including graphene, carbon nanotubes, nanosheets, and biomass-derived amorphous carbon (Li et al., 2016; de Medeiros et al., 2020; Ghani et al., 2021). Graphene has excellent electron-transport properties and low electrical conductivity, which reduces the recombination of photogenerated carriers. It has strong absorption in both ultraviolet and visible light regions, resulting in improved utilization of sunlight. Yao et al. reported the esterification catalyzed by commercial graphene oxide, and the catalytic effect was not obvious (the ester yield: 33%), which may be due to the weak electron-hole oxidation ability (Yao et al., 2022). Hussain et al. prepared ZnO/Ni-SBA-16@GO composite photocatalyst for conversion of waste edible oil into biodiesel with good activity under 500 W Xe lamp irradiation, and the conversion rate was as high as 98% (Athar Hussain et al., 2022). Similar to graphene-semiconductor materials, as doped with graphene on the semiconductor, VB above would produce donor level (introduction of C 2p orbital), leading to the semiconductor VB upshift and narrow band gap. The semiconductors are sensitized to improve their optical absorption ability due to the narrow bandgap of graphene. Graphitic carbon nitride (g-C_3_N_4_, E_g_ = 2.7 eV), as a low-cost, and non-toxic photocatalyst, has shortcomings like small specific surface area, poor long-wavelength light absorption, low charge transfer efficiency, and high photogenerated electron-hole pair recombination rate, which limits its performance. Ghani et al. used graphene carbon nitride composite (SrTiO_3_/g-C_3_N_4_) to catalyze frying waste oil and methanol for preparing biodiesel, and the obtained yield was up to 96% (Ghani et al., 2021). Therefore, graphite-phase carbon nitride is usually applied to photoreaction in the form of composite materials. The above composite photocatalysis shows good photocatalytic performance in the process of catalytic production of biodiesel, and the quality of biodiesel meets the American Society for Testing and Materials (ASTM) standards. Although carbonaceous photocatalytic materials have been extensively studied, they still face three major challenges. 1) The photocatalytic performance of carbon-based materials is closely related to the preparation process, defect degree, and functional materials. 2) The interface charge transfer mechanism is not thorough enough. 3) Carbon-based materials have relatively high emissivity (about 0.85) due to its high surface reflection.

In addition to the above types of photocatalytic materials, other materials (e.g., MOFs, MXenes, polypyrroles, and polyanilines) exhibit the great potential to be used as photocatalysts for the production of biodiesel with high performance (He et al., 2020; Liu et al., 2021; Qian et al., 2021; Song et al., 2022), while much more efforts should be made to simplify the catalyst preparation procedures, facilitate large-scale production processes, and so on.

## Strategies for Improving Photocatalyst Performance

In this section, several typical modification strategies to improve the photocatalytic performance of photocatalysts are discussed. The catalytic performance and solar energy conversion efficiency of photocatalysts can be improved from three aspects: light absorption, photogenerated carrier separation, and interfacial reaction. Visible and infrared light accounts for 96%, ultraviolet light accounts for only 4% in sunlight, so it is necessary to enhance the light absorption of photocatalytic materials. The light absorption properties of photocatalysts can be modified by band adjustment, morphology control, and sensitization. A narrow band gap is beneficial to light absorption. Metal and non-metal doping is an effective strategy to change the band gap width and band edge position of the materials (Li et al., 2013; Li et al., 2019a). In addition, the photocatalytic materials can be prepared with different morphologies, such as multilayer nanosheets, multilayer hollow, and nano-helical column structures, which can promote the multiple reflection absorption of sunlight (de Medeiros et al., 2020; Ghani et al., 2021). The most direct way is to directly add dyes, quantum dots, and other sensitizers to photocatalytic materials, which can also enhance light absorption (Karimi Estahbanati et al., 2020).

The generated photogenerated carriers need to be transmitted to the interface to participate in the reaction. During this process, photogenerated carriers are easy to contact organic compounds. The construction of the local electric field, morphology control, and interface modification strategies can be used to promote the separation and transmission of photogenerated carriers (Wang L et al., 2018; Hassani et al., 2021; Nishiyama et al., 2021). The heterojunction is the interface region formed by the contact of two different semiconductors, which produces electron and hole concentration differences at the interface, thus forming a heterojunction electric field that can promote the separation of carriers. The morphology of photocatalytic materials affects their photocatalytic performance, and the exposure of different crystal faces may affect the interface reaction process (Karimi Estahbanati et al., 2020). By adjusting the morphology of catalysts, including nanotubes, wires and rods, the excellent charge transferability is attributed to their high crystallization and oriented structure. The interface modification strategy can also reduce the interface recombination of photogenerated carriers, thereby improving the charge separation efficiency.

In the photocatalytic production of biodiesel, if the photogenerated carriers cannot be captured by oil and alcohol, the recombination and accumulation of carriers will be caused. Firstly, the reactants should be adsorbed on the active site of the photocatalyst, so the reactants can be strongly adsorbed by oxygen vacancies on the catalyst (Li et al., 2015b). The added cocatalyst can reduce the accumulation and recombination of carriers, and further introduce other modification layers between the modification layer and the semiconductor layer, which may be beneficial to the separation and transmission of carriers (Hassani et al., 2021; Nishiyama et al., 2021). Moreover, stirring can promote the adsorption of reactants and the desorption of products, which is conducive to the capture of photogenerated carriers by reactants, thereby speeding up the reaction (Guo et al., 2021).

## Conclusion and Outlook

Biodiesel presents a promising alternative to fossil fuels to provide energy to the world. The photocatalytic production of biodiesel under mild conditions holds significant potential in enabling such a transition. Compared to conventional thermal-driven catalysis, photo-driven catalysis has the advantages of green, environmental protection, and energy-saving. Recent developments in the photo-driven catalytic production of biodiesel were summarized from three aspects (trans)esterification mechanism, photocatalytic type, and modification strategy. Photocatalysts appeared to enhance light absorption by adjusting the band gap, morphology control, and adding sensitizers. Local electric field, morphology control, and interface modification strategies were constructed to promote the separation and transmission of photogenerated carriers. To promote the interfacial reaction, the introduction of oxygen on the photocatalyst to enhance the adsorption of reactants, and co-catalysts and stirring were added to promote the reaction, thereby reducing the accumulation and recombination of photogenerated carriers.

There are still some problems to be solved in the production of biodiesel by photo-driven catalysis. First of all, mechanisms of photo-catalytic (trans)esterification have not been systematically studied due to few studies on this topic. The photocatalytic production of biodiesel shows excellent performance in the laboratory, but most of the photocatalysts with excellent catalytic performance generally contain expensive noble metals, so the design and preparation of efficient non-noble metal or non-metallic materials are of great significance for photo-driven catalysis. In addition, the industrial application of photocatalytic production of biodiesel is still a breakthrough new field. At present, photocatalytic equipment has not been standardized and there is no complete set of equipment. Therefore, the real goal, in the next few years, is to transfer these catalytic processes from laboratory to industrial scale. Overall, photo-driven catalytic biodiesel production is still in its infancy, yet it opens a new door for the green production of biofuels. The industrialization of photo-driven catalytic biodiesel production needs the continuous efforts of researchers in this field.
